# Development of novel vaccine candidates and challenge models for *Plasmodium vivax*

**DOI:** 10.1186/1475-2875-13-S1-P4

**Published:** 2014-09-22

**Authors:** Eduardo Alves, Ahmed Salman, Chris Janse, Shahid Khan, Adrian Hill, Arturo Reyes-Sandoval

**Affiliations:** 1 Oxford University, Oxford, UK; 2 Leiden Malaria Research Group, Leiden, The Netherlands

## 

*Plasmodium vivax* is the most widely distributed human malaria parasite in the world and despite nearly 2.5 billion people living at risk, only four vaccines have been assessed in phase I clinical trials and only one has progressed to a phase II trial showing no sterile efficacy. We started to develop new challenge models to assess the efficacy of several new *P. vivax* pre-erythrocytic vaccine candidates (PVX_091700; PVX_121950; PVX_084090; PVX_099035; PVX_095375; PVX_000975; PVX_003665; PVX_000810) in mice. Our model is based on creating mutant *P. berghei* (rodent malaria) lines expressing *P. vivax* antigens through a new method called “Gene Insertion/Marker out” (GIMO). In addition, we are cloning the *P. vivax* pre-erythrocytic vaccine candidates in recombinant chimpanzee adenovirus (ChAd) and modified vaccine Ankara (MVA) vectors. Upon generation of transgenic parasites and recombinant viruses, a faster assessment to determinate the efficacy of all new *P. vivax* vaccine candidates can be achieved by using prime/boost immunization regimens followed by a challenge with corresponding transgenic chimera parasites.

